# Supplementary material for: Condensin loop extrusion properties, roadblocks, and role in homology search during recombination in S. cerevisiae
Source: EMBO J. 2026 Mar 23;45(9):3124–55. doi: 10.1038/s44318-026-00748-6 (PMC13144730; doi:10.1038/s44318-026-00748-6)
Supplement: Supplementary file 7 — Expanded View Figures [file 44318_2026_748_MOESM7_ESM.pdf]

Expanded View Figures

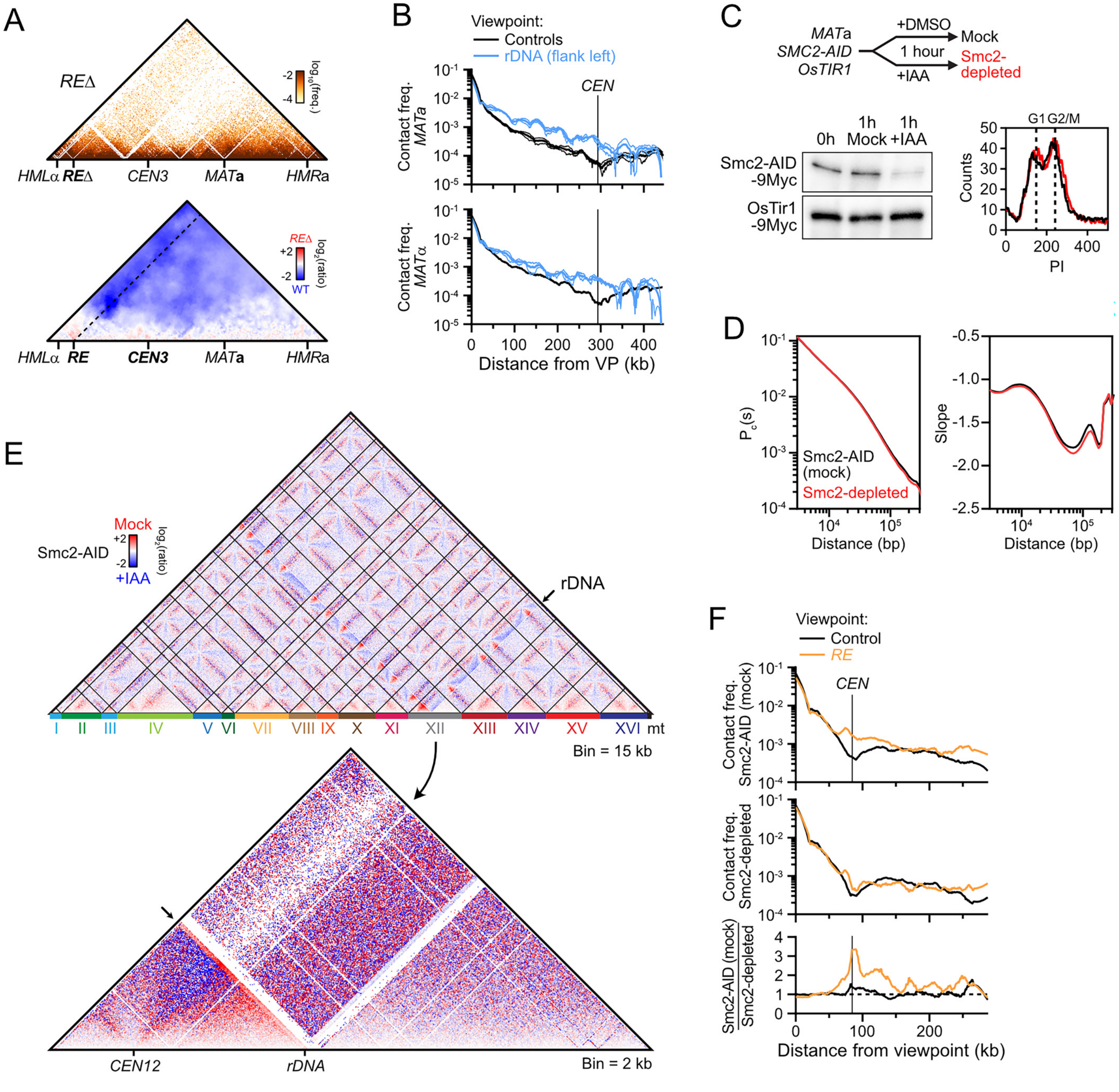

Figure EV1. Condensin- and *RE*-dependent contact stripes in *MATa* cells.

(related to Fig. 1). (**A**) Top: Hi-C contact map of chr. III in a *RE*-deleted *MATa* strain (APY1548). Bin: 1 kb. Bottom: Ratio map over a WT strain. Data show $n = 1$ biological replicate. (**B**) 4C-like contact profile of the left *rDNA*-flanking region (blue) and of the average of 6 control sites (black) as viewpoints in *MATa* and *MATα* cells, from Hi-C data in Fig. 1A. (**C**) Smc2 depletion scheme, Western blot validation, and FACS profiles of Smc2-AID (mock) and Smc2-depleted cells. (**D**) Probability of contact as a function of the genomic distance (Pc(s)) and its derivative in Smc2-AID (mock) and Smc2-depleted cells. (**E**) Ratio maps of the whole genome (top) and chr. XII (bottom) in cells proficient and deficient for condensin. (**F**) Top: 4C-like contact profiles of the *RE* and of the average of 6 control sites in Smc2-AID-tagged (mock) and Smc2-depleted samples, from Hi-C data in Fig. 1C. Bottom: Ratio of *RE* and control 4C-like profiles of Smc2-AID (mock) over Smc2-depleted samples. Data show $n = 1$ biological replicate.

© The Author(s)

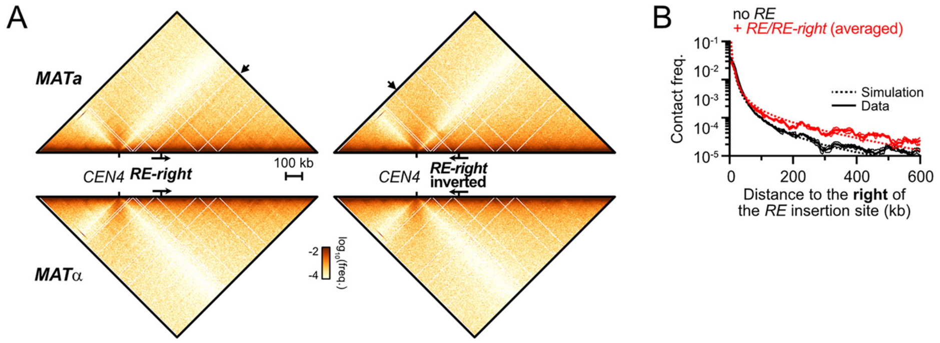

Figure EV2. Condensin loop extrusion properties.

(related to Fig. 2). (**A**) Top: Hi-C contact maps of chr. IV in *MAT*a and *MAT*α cells bearing the *RE-right* construct at position 680 kb either in the forward (APY1850 and APY1852) or inverted (APY2058 and APY2060) orientation. Hi-C maps are binned at 5 kb. Data show $n = 1$ biological replicate. (**B**) Observed and simulated 4C-like profiles using chr. IV 680 kb as a viewpoint, either unmodified ("no *RE*" black), or upon insertion of the *RE* or the *RE-right* constructs (data averaged). From data in Fig. 2C. The ratio of the "+*RE*" profiles over the "no *RE*" profiles gives rise to the normalized data presented in Fig. 2G.

 © The Author(s)

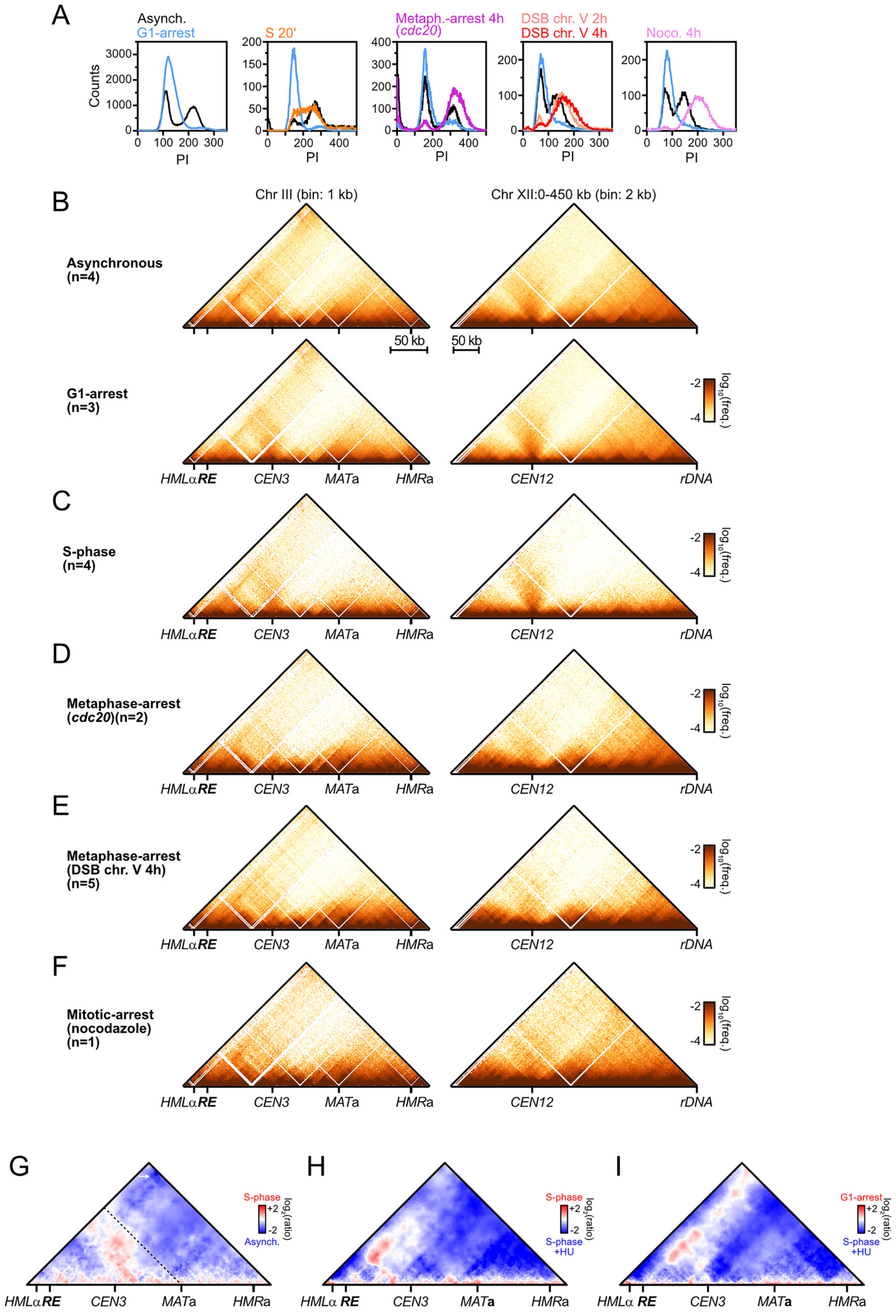
A
Asynch.
G1-arrest
S 20'
Metaph.-arrest 4h (cdc20)
DSB chr. V 2h
DSB chr. V 4h
Noco. 4h
Counts
PI
B
Chr III (bin: 1 kb)
Chr XII:0-450 kb (bin: 2 kb)
Asynchronous (n=4)
G1-arrest (n=3)
50 kb
HMLα RE
CEN3
MATa
HMRa
CEN12
rDNA
log10(freq.)
C
S-phase (n=4)
D
Metaphase-arrest (cdc20)(n=2)
E
Metaphase-arrest (DSB chr. V 4h) (n=5)
F
Mitotic-arrest (nocodazole) (n=1)
G
S-phase
Asynch.
log2(ratio)
H
S-phase +HU
I
G1-arrest

Figure EV3. Regulation of loop extrusion by condensin across the cell cycle.

(related to Fig. 3). (**A**) FACS profiles of the different cell-cycle stages studied here. (**B–F**) Hi-C maps of chr. III and chr. XII:0-450 kb (**A**) upon G1-arrest (APY266), (**B**) during S-phase (APY539 and APY607, merged), (**C**) upon metaphase-arrest due to *CDC20* repression (APY537), (**D**) upon DDC-induced metaphase-arrest due to formation of a single unrepairable HO-induced DSB on chr. V (APY266), and (**E**) upon mitotic-arrest in the presence of nocodazole (APY266). All cells are *MAT*a. The number of biological replicates (n) is indicated in each panel. Bin: 1 kb (chr. III) or 2 kb (chr. XII). (**G**) Ratio map highlighting the changes to chr. III structure in S-phase vs. asynchronous *MAT*a cells. From data in (**B**, **C**). (**H**) Same as (**G**) in untreated vs. HU-treated cells in S-phase. From data in (Jeppsson et al, 2022). (**I**) Same as (**G**) in G1-arrested vs HU-treated S-phase cells. From data in (Jeppsson et al, 2022).

 © The Author(s)

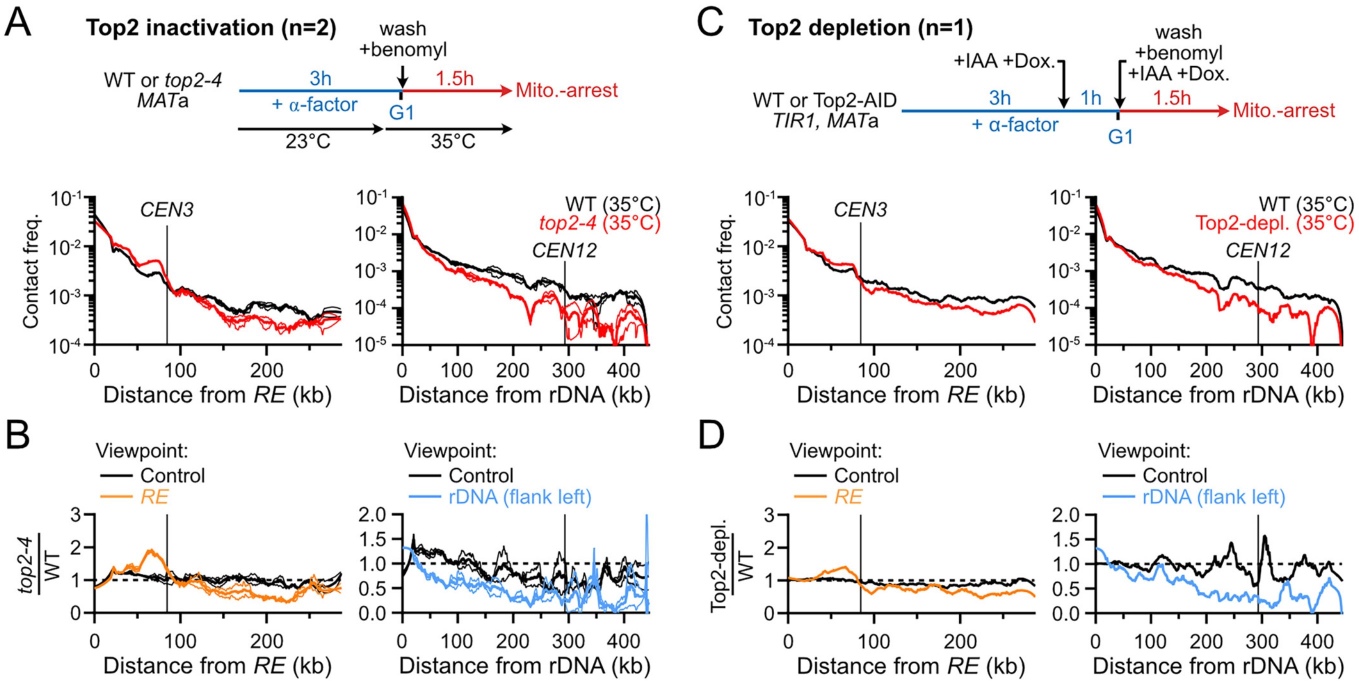

Figure EV4. Loop extrusion by condensin is compromised in Top2-deficient cells.

(**A**) 4C-like profiles of the *RE* (left) and the rDNA (right) and their cognate control sites in a mitotic WT and *top2-4* strains after 1.5 h at restrictive temperature. Data show mean ± SEM of $n = 2$ biological replicates. (**B**) Ratio of 4C-like profiles for the *RE*, *rDNA* and their cognate controls sites in *top2-4* over WT cells. Data show mean ± SEM of $n = 2$ biological replicates. (**C**) 4C-like profiles of the *RE* (left) and the rDNA (right) and their cognate control sites in mitotic WT and Top2-depleted strains. Data show $n = 1$ biological replicate. (**D**) Ratio of 4C-like profiles for the *RE*, *rDNA* and their cognate controls sites in WT and Top2-depleted cells. Data show $n = 1$ biological replicate. (**A–D**) All data are from (Jeppsson et al, 2024).

© The Author(s)

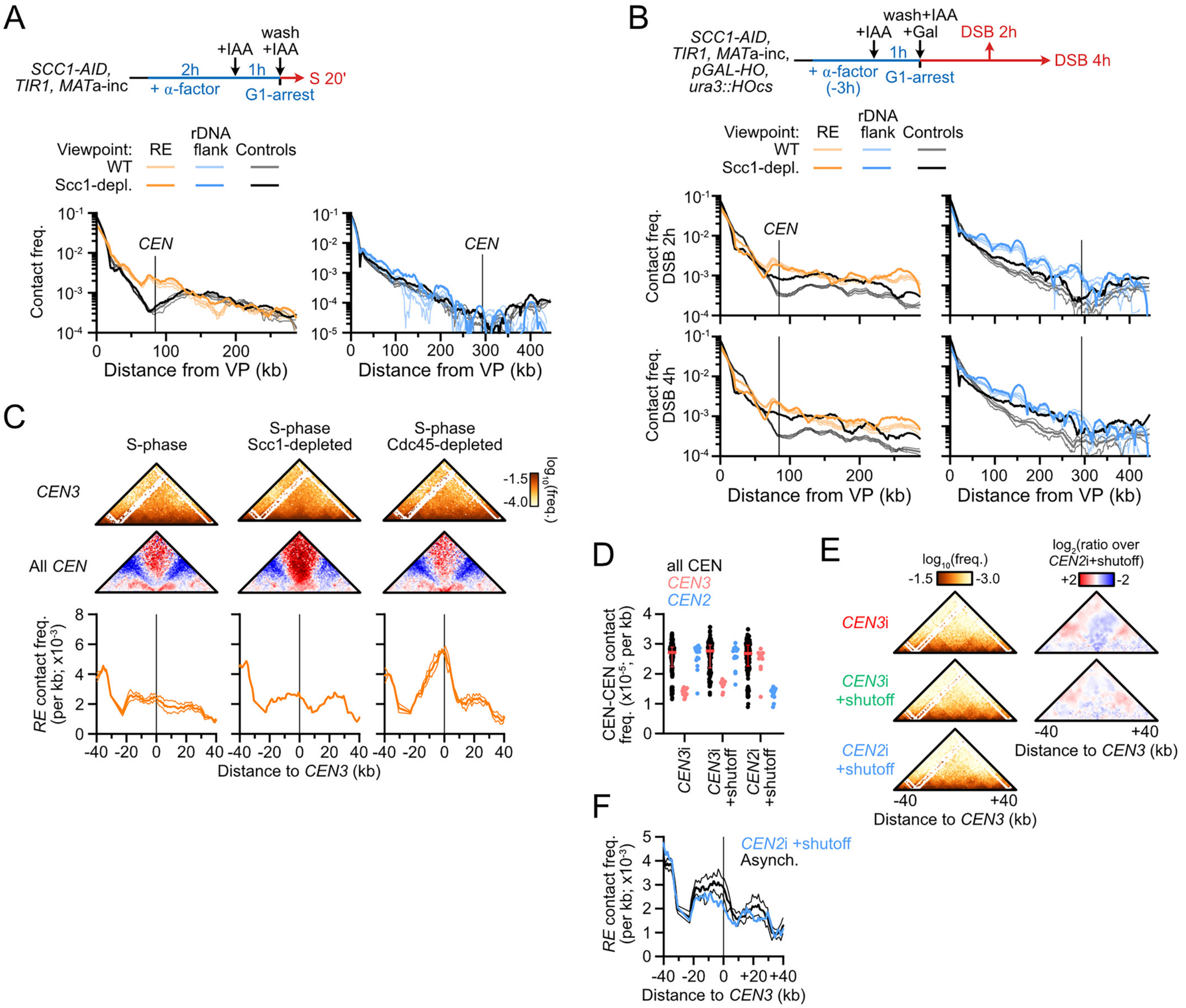

Figure EV5. The centromere stalls condensin translocation in a kinetochore-dependent manner.

(related to Fig. 4). (**A**) Loss of Scc1 does not rescue condensin-mediated loop extrusion in S-phase. Top: Scheme for Scc1-AID depletion prior to S-phase release. Bottom: 4C-like profiles at the *RE* and the left *rDNA*-flanking region and their corresponding control sites in WT and Scc1-depleted cells. $n = 2$ and 1 biological replicates, respectively. Data are from (D'Asaro et al, 2025). (**B**) Loss of Scc1 does not affect condensin-mediated loop extrusion in metaphase cells. Same as (**A**), but with Hi-C performed in cells arrested in metaphase upon formation of an unrepairable DSB on chr. V. Note the elevated baseline, particularly at 4 h post-DSB induction. $n = 4$ and 1 biological replicates for WT and Scc1-depleted cells, respectively. Data are from (Dumont et al, 2024). (**C**) Condensin roadblock at *CEN3* in S-phase in WT, Scc1-depleted, and Cdc45-depleted cells. Top: Hi-C maps of the *CEN3*-surrounding region. Middle: Aggregated contact maps of all centromeres. Bottom: *RE*-contact stripes. $n = 2$, 1, and 1 biological replicates for WT, Scc1-depleted, and Cdc45-depleted cells, respectively. Data are from (D'Asaro et al, 2025). (**D**) Inter-chromosomal contact frequency between all centromeres (black), between *CEN3* and other centromeres (pink), and between *CEN2* and other centromeres (blue) following transcription-mediated *CEN3* or *CEN2* inactivation. Each point represents a CEN-CEN contact frequency. Bars show median ± inter-quartile range. Data are from Fig. 4E, with each condition corresponding to $n = 1$ biological replicate. (**E**) Left: Hi-C contact maps of the *CEN3*-surrounding region (bin: 1 kb). Right: ratio maps of the *CEN3*-surrounding region in *CEN3*-inactivated cells over control *CEN2*-inactivated cells. (**F**) 4C-like profiles with the *RE* as a viewpoint in WT and *CEN2*-inactivated strains. Data show mean ± SEM of $n = 4$ and 1 biological replicates, respectively.

 © The Author(s)

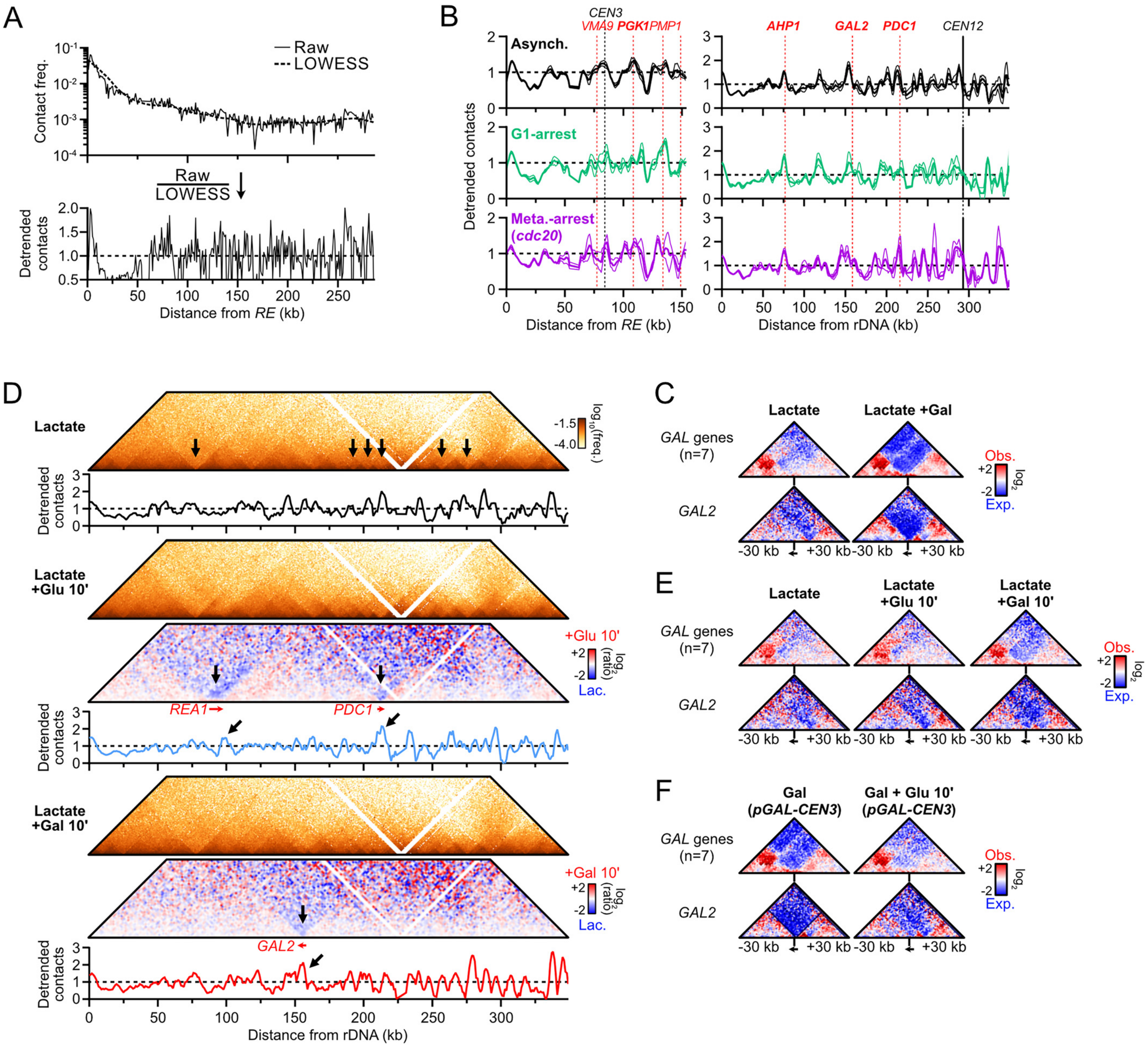

Figure EV6. Highly transcribed RNA PolII-dependent genes stall condensin translocation.

(related to Fig. 5). (**A**) Rationale for raw contact detrending over the LOWESS regression. (**B**) Detrended *RE* and *rDNA*-flanking contacts in asynchronous, G1-arrested, and metaphase-arrested cells grown in the presence of galactose and in the absence of glucose. Data are the same as in Figs. 1A and 3A,C. Highly transcribed genes present at the major peaks are indicated. (**C**) Observed over expected ratio maps aggregated at all *GAL* genes (top) and at *GAL2* (bottom) in galactose- and glucose-containing media. (**D**) Correspondence between high transcription (visible as discrete borders in the Hi-C map) and condensin loop extrusion pausing in lactate media and upon glucose or galactose addition for 10 min. $n = 1$ biological replicate each. (**E**) As in (**C**), from data in (**D**). (**F**) As in (**C**), upon glucose addition in galactose-containing media. From data in Figs. 4E and 5D.

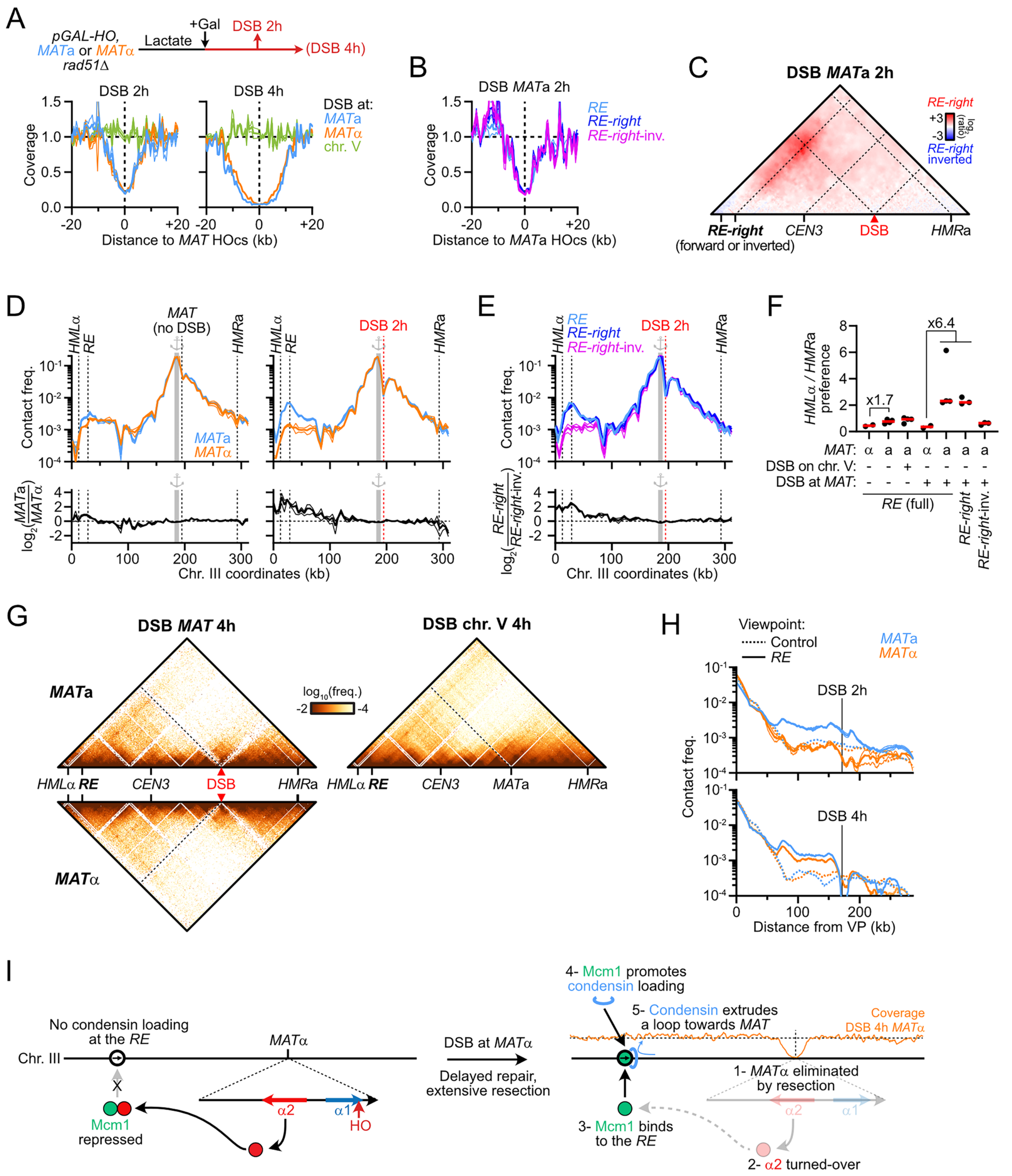

A
pGAL-HO, MATa or MATα rad51Δ
Lactate
+Gal
DSB 2h
(DSB 4h)
DSB 2h
DSB 4h
DSB at: MATa MATα chr. V
Coverage
Distance to MAT HOcs (kb)
B
DSB MATa 2h
RE
RE-right
RE-right-inv.
Distance to MATa HOcs (kb)
C
DSB MATa 2h
RE-right
RE-right inverted
RE-right (forward or inverted)
CEN3
DSB
HMRa
D
MAT (no DSB)
HMLα
RE
HMRa
DSB 2h
Contact freq.
MATa
MATα
Chr. III coordinates (kb)
E
DSB 2h
F
HMLα / HMRa preference
x1.7
x6.4
MAT:
DSB on chr. V:
DSB at MAT:
RE (full)
RE-right
RE-right-inv.
G
DSB MAT 4h
DSB chr. V 4h
MATa
MATα
log10(freq.)
HMLα RE CEN3 DSB HMRa
HMLα RE CEN3 MATa HMRa
H
Viewpoint:
Control
RE
MATa
MATα
DSB 2h
DSB 4h
Contact freq.
Distance from VP (kb)
I
No condensin loading at the RE
Chr. III
MATα
Mcm1 repressed
α2
α1
HO
DSB at MATα
Delayed repair, extensive resection
4- Mcm1 promotes condensin loading
5- Condensin extrudes a loop towards MAT
Coverage DSB 4h MATα
1- MATα eliminated by resection
3- Mcm1 binds to the RE
2- α2 turned-over

 © The Author(s)

Figure EV7. DSB formation at *MAT*a blocks condensin translocation and creates a *RE*-DSB loop.

(related to Fig. 6). (**A**) Coverage from Hi-C reads at 2 and 4 h post-DSB induction at *MAT* in *MAT*a and *MAT*α cells. Control cells with a DSB on chr. V show no loss of coverage at *MAT*. From data in Fig. 6A. (**B**) Coverage from Hi-C reads at 2 h post-DSB induction at *MAT*a in cells with *RE* variants. From data in Fig. 6D. (**C**) Ratio map of cells with the *RE-right* over the *RE-right*-inverted construct 2 h post-DSB induction at *MAT*a. From data in Fig. 6D. (**D**) Top: 4C-like profiles with the 10 kb region left of *MAT* as a viewpoint in asynchronous cells and in cells 2 h post-DSB induction. Bottom: Log_2 ratio of profiles in *MAT*a over *MAT*α cells showing specific enrichment of contact between *MAT*a and the *HML*α-*RE* interval. From data in Figs. 1A and 6A. (**E**) Top: 4C-like profiles with the 10 kb region left of *MAT* as a viewpoint in *MAT*a cells bearing different *RE* variants 2 h post-DSB induction. Bottom: Log_2 ratio of 4C profiles in *RE-right* over *RE-right*-inverted-containing cells. From data in Fig. 6D. (**F**) Quantification of the preference for *MAT* interaction with *HML* vs. *HMR*. Black data points show individual biological replicates (*n*), and the red bar shows the median. (**G**) Hi-C contact maps in *MAT*a and *MAT*α *rad51*Δ cells (APY1267 and APY1264, respectively) at 4 h post-DSB induction at *MAT*. Data show $n = 1$ biological replicate each. A *MAT*a cells with an unrepairable DSB on chr. V (APY266) is shown for comparison 2 h post-DSB induction ($n = 5$ biological replicates). Bin: 1 kb. (**H**) 4C-like profiles with the *RE* (or 6 control sites) as a viewpoint, from data in (**G**) and Fig. 6A. Data show mean ± SEM. (**I**) Model for the reactivation of condensin-mediated loop extrusion from the *RE* and establishment of a *RE*-DSB loop upon defective repair of a DSB at *MAT*α.

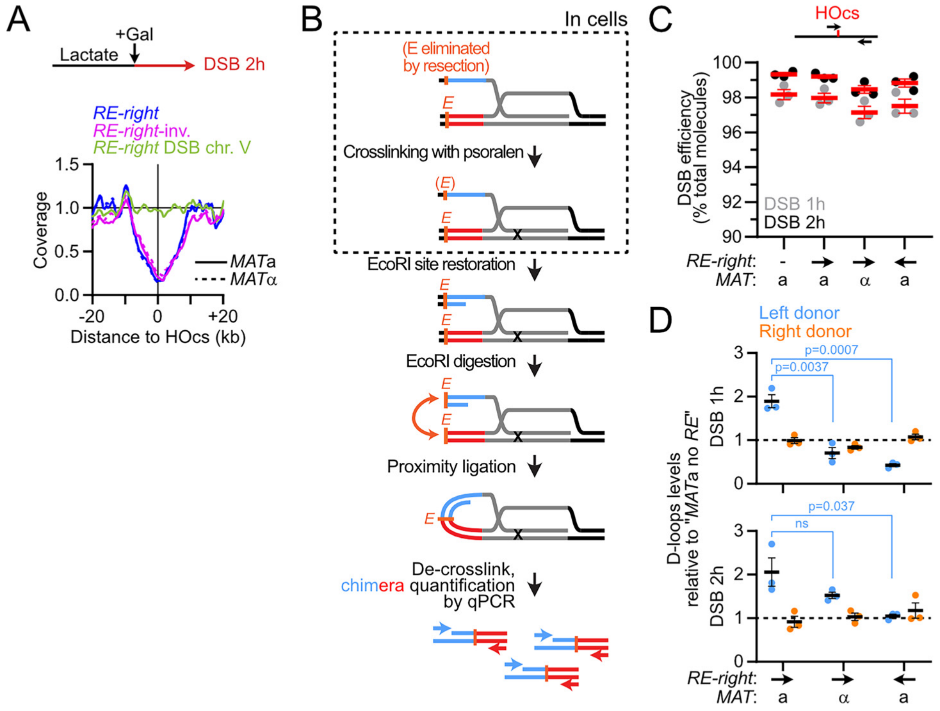

Figure EV8. The *RE*-DSB loop is portable and promotes *RE*-proximal homology search.

(related to Fig. 7). (**A**) Coverage from Hi-C reads at 2 h post-DSB induction at the HOcs on chr. IV in *MAT*a and *MAT*α cells bearing the *RE-right* in forward or inverted orientation. Control *MAT*a cells with a DSB on chr. V show no loss of coverage at that site. From data in Fig. 7A. (**B**) Rationale of the D-loop Capture assay. (**C**) Quantification of DSB formation at 1 and 2 h post-induction. Data points show individual biological replicates (*n*). Mean ± SEM are shown in red. (**D**) D-loops levels expressed relatively to that measured in the *MAT*a strain without *RE* assayed in parallel. From data in Fig. 7D. Data show individual biological replicates (*n*) as well as mean ± SEM. *P* values were obtained using a Student *t* test. None of the comparisons for the right donor are significant.

 © The Author(s)
